# Facial Morphology Assessment in Brazilian Children With Congenital Zika Virus Syndrome: A Cross‐Sectional Anthropometric Study

**DOI:** 10.1155/ijod/9978691

**Published:** 2026-09-28

**Authors:** Dhaniel Anderson Olimpio Barbosa, Mariana Oliveira Pinto de Sousa, Maria Lúcia Moreira da Rocha, Cláudio Roberto Tavares Pereira Filho, Maria Cláudia de Freitas Lima, Ellaine Doris Fernandes Carvalho, Maysa Luna de Souza, Paulo Goberlânio de Barros Silva, Paulo Tárcio Aded da Silva, Jonathan Francisco de Melo Silva, Isabella Fernandes Carvalho, Phillipe Nogueira Barbosa Alencar

**Affiliations:** ^1^ Department of Dentistry, Christus University Center, Fortaleza, Ceará, Brazil, unichristus.edu.br

**Keywords:** congenital abnormalities, microcephaly, Zika virus

## Abstract

**Objectives:**

This study aimed to conduct an anthropometric analysis of children with microcephaly caused by Zika virus infection and to compare results with those of a control group.

**Materials and Methods:**

The study included 12 children with congenital microcephaly (experimental group) and 15 normosystemic children (control group), all aged 4–5 years. Eight anthropometric facial points were marked to assess the upper, middle, and lower thirds of the face.

**Statistics:**

To assess normality, the Shapiro–Wilk test was used, followed by the Student’s *t*‐test to compare groups at the 95% confidence level.

**Results:**

Orofacial anthropometric measurements showed that upper lip measurements and upper‐to‐lower lip ratios were higher in the experimental group than in the control group; however, only upper lip variables showed statistically significant differences (*p*‐values < 0.05). Conclusion: Overall, the study found that the experimental group had a significantly larger upper lip than the control group. While measurements for the lower third of the face were also higher in the experimental group, these differences did not reach statistical significance.

## 1. Introduction

Zika virus (ZIKV) is an arbovirus of the *Flaviviridae* family. It is primarily transmitted by *Aedes* mosquitoes. First identified in 1947 in the Zika Forest in Uganda, the virus remained relatively obscure. Large outbreaks occurred decades later in the Pacific region and then in the Americas. In 2015, Brazil became the epicenter of the epidemic. The country registered hundreds of thousands of suspected infections and drew global attention to the severe consequences of prenatal exposure to ZIKV [1, 2].

One of the most significant outcomes linked to vertical transmission of ZIKV is microcephaly. This neurological condition has a multifactorial origin and is marked by impaired brain development and reduced head circumference compared to peers. Affected children may have delayed cognitive development, abnormal motor coordination, speech impairments, epilepsy, visual and hearing deficits, and cerebral palsy. Studies have shown that ZIKV can cross the placental barrier and disrupt neurogenesis and fetal development. This reinforces its teratogenic potential [3, 4].

Transmission of ZIKV from mother to fetus is linked to microcephaly and other brain abnormalities. This relationship has highlighted the urgent need to study the virus’s teratogenic effects and mechanisms. Studies in rats have shown that ZIKV readily crosses the placental barrier and impairs fetal development [3, 4].

Microcephaly is not the only issue linked to ZIKV. A wide range of clinical signs has been described, leading to recognition of congenital Zika virus syndrome (CZS). This condition includes neurological impairment, eye issues, musculoskeletal changes, hypotonia, arthrogryposis, and sensory deficits. These issues may persist throughout childhood and affect overall development [5, 6]. Recent studies show that children with CZS often have complex limitations that reach beyond the central nervous system. These limitations affect feeding, communication, and orofacial functions. They can also have long‐term consequences for craniofacial growth and development [7, 8].

Recent evidence suggests that children with CZS may have lasting craniofacial growth issues during postnatal development [9–11]. Studies of facial growth in both affected and healthy groups show that functional factors—such as muscle tone, feeding, and chewing patterns—play a critical role in shaping facial features over time [12]. These findings underscore the need to investigate facial dimensions in children with CZS within a broader developmental and functional context.

Recent advances in craniofacial analysis include the use of three‐dimensional (3D) imaging technologies, such as facial scanning and 3D cephalometric analysis, which allow highly accurate morphometric evaluations and the development of normative craniofacial databases [13]. Although these technologies have expanded the possibilities for craniofacial assessment, direct anthropometry remains a reliable and widely accepted method in craniofacial research. This approach offers important practical advantages, including low cost, feasibility in field conditions, absence of ionizing radiation, and suitability for pediatric populations and individuals with special healthcare needs.

Despite the increase in studies on CZS, quantitative assessments of facial morphology using objective anthropometric methods remain rare. Most research has focused on neurological, eye, and dental issues. This leaves a gap in the study of craniofacial patterns in this group. This study aimed to evaluate facial dimensions in children with CZS using standardized anthropometric measurements. Understanding these craniofacial traits may improve clinical assessment. It can also support early interventions for feeding, speech, and breathing in children with CZS.

## 2. Materials and Methods

### 2.1. Study Design and Ethical Approval

The study followed the ethical principles for research involving human subjects as outlined in Resolution 466/12 of the Brazilian National Health Council. The Research Ethics Committee of Centro Universitário Christus (Unichristus) approved the study under protocol number 1881086 in December 2016.

### 2.2. Participants

Twelve children aged 4–5 years with CZS were included in the experimental group. Fifteen children of similar age, without CZS or other major systemic conditions, were included in the control group. Children with CZS were referred from medical services to the Unichristus School of Dentistry Clinic. Control group participants were recruited from patients attending the Pediatric Dentistry Clinic at the same institution.

Due to the rarity of congenital Zika syndrome and the limited number of eligible children diagnosed between 2015 and 2016, a convenience sample was used, including all participants who met the inclusion criteria during the study period. Although the groups were not perfectly balanced by sex, they showed similar proportions. Given the narrow age range (4–5 years), the influence of sexual dimorphism on facial measurements is expected to be minimal [9].

### 2.3. Sample Size

Sample size estimation was based on the incidence of delayed tooth eruption reported by Carvalho et al. [14], which was significantly higher in children with ZIKV compared to controls (60.0% vs. 3.3%). Based on this parameter, a minimum of 15 participants per group was estimated to achieve 90% statistical power with a 95% confidence level (Fleiss method with continuity correction). However, three children from the CZS group were excluded due to sample loss, resulting in a final sample of 12 children with CZS and 15 children in the control group.

### 2.4. Anthropometric Measurements

Anthropometric measurements were performed according to standardized procedures described by Farkas and Deutsch [15]. The measurements were taken using a digital caliper (Carbon Fiber Composites Digital Caliper), an instrument designed to measure linear distances between two points.

Researchers palpated the orofacial anthropometric landmarks before collecting measurements to ensure accurate localization. Before taking measurements, the caliper was zeroed by closing it fully and pressing the zeroing button until the dial read 0.00.

Orofacial anthropometric measurements were taken in the frontal view and recorded in millimeters for the collection protocol (Figures 1 and 2).

**Figure 1:**
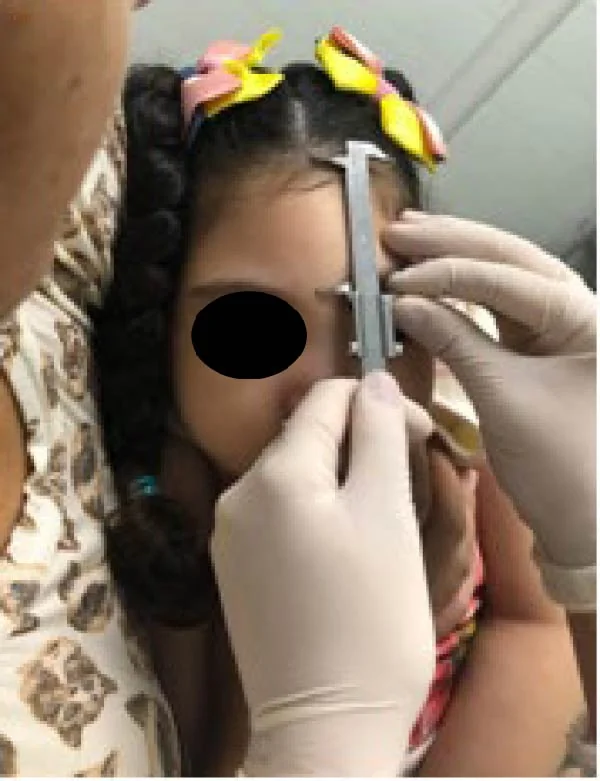
Measurement of the upper third of the face.

**Figure 2:**
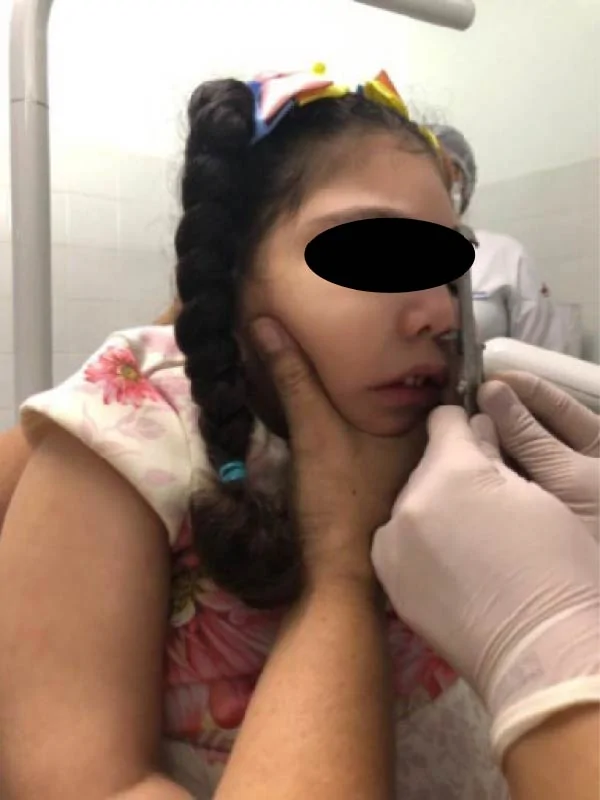
Measurement of the middle third of the face.

The points were carefully marked on the children’s faces (Figure 3) using a black Make B eye pencil.

**Figure 3:**
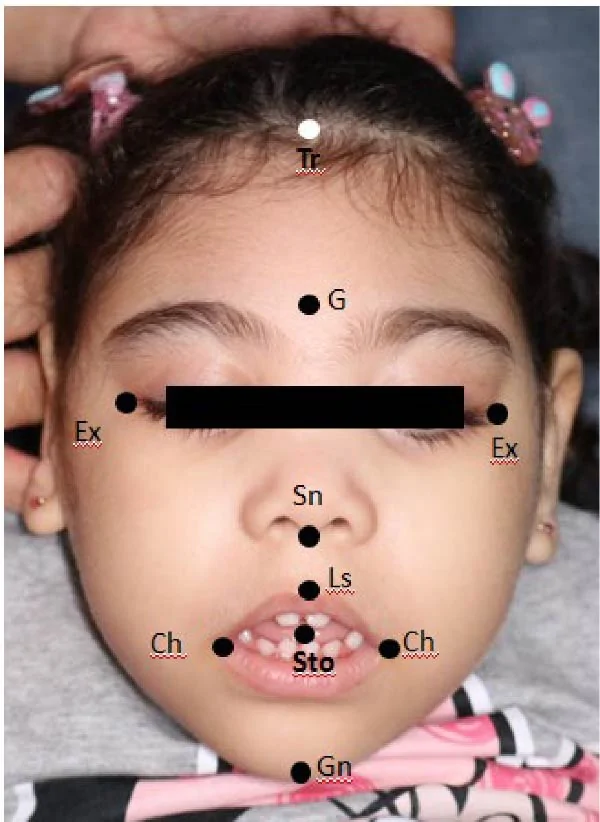
Anthropometric points. *Glabella* (*g*): The most prominent point on the midline between the eyebrows, located on the frontal bone. *Trichion* (*tr*): The point located at the hairline, on the midline of the forehead. *Gnathion* (*gn*): The lower median point of the lower border of the mandible. *External corner of the eye* (*ex*): Located at the lateral commissure of the eyelids and medial to the external corner of the bony eye. *Subnasal* (*sn*): The median point at the angle of the base of the columella, where the lower edge of the nasal septum meets the surface of the upper lip. *Upper lip* (*ls*): The median point located on the red line of the upper lip. *Stomalium* (*sto*): An imaginary point at the intersection of the median vertical line of the face, which connects the *trichion* (*tr*), *subnasal* (*sn*), *and gnathion* (*gn*), and the horizontal line of the mouth’s rima, when the lips are slightly closed and the teeth are occluded. *Cheilon* (*ch*): The point located at the commissure of the lips. Height of the upper lip (from *subnasal* to *stomium* or sn‐sto). Height of the lower lip (from *stomium* to *gnathion* or sto‐gn). Philtrum height (from *subnasal* to *upper labial* or sn‐ls). Height of the upper third of the face (from *trichion* to *glabella* or tr‐g). Height of the middle third of the face (from *glabella* to *subnasal* or g‐sn). Height of the lower third of the face (from *subnasal* to *gnathion* or sn‐gn). Distance between the outer corner of the eye and the cheilion on the left side of the face (ex‐ch).

After the anthropometric measurements were established, the following orofacial proportions were analyzed: the proportion between the upper lip (sn‐sto) and the lower lip (sto‐gn); between the upper third of the face (tr‐g) and the middle third (g‐sn); and between the middle third of the face (g‐sn) and the lower third (sn‐gn). The anthropometric measurements are illustrated in Figure 4.

**Figure 4:**
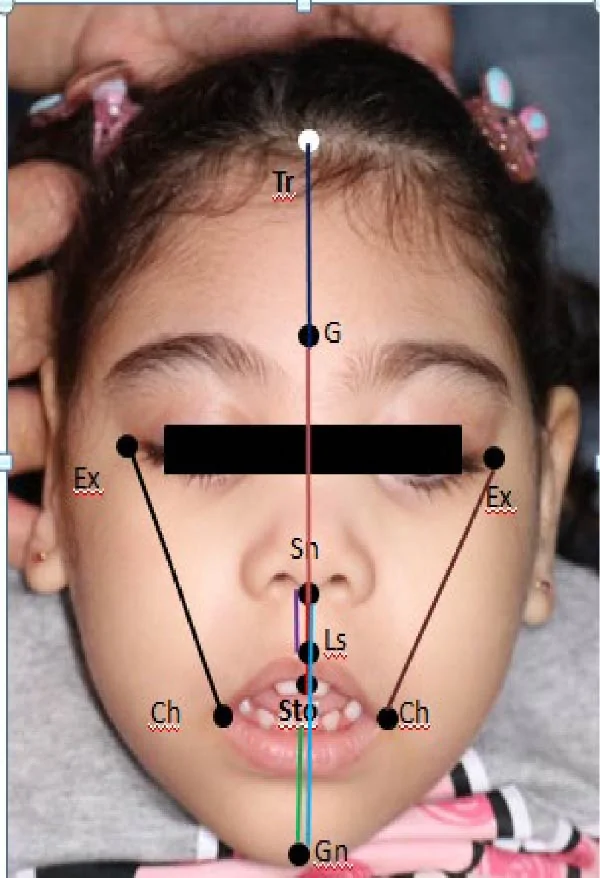
Tracing of the anthropometric measurements.

Individual orofacial anthropometric measurements and calculated facial proportions for children with congenital Zika virus syndrome and normosystemic children are provided in the Supporting Information S1–S4.

### 2.5. Examiner Reliability

Two independent examiners performed all measurements twice. The arithmetic mean of the measurements was used as the final value for each variable.

### 2.6. Statistical Analysis

Data were expressed as mean ± standard deviation and analyzed for normality using the Shapiro–Wilk test. Comparisons were made using Student’s *t*‐test (parametric data). All analyses were performed at a 95% confidence level using the Statistical Package for the Social Sciences (SPSS version 20.0 for Windows).

## 3. Results

After analyzing the collected data, the arithmetic means and standard deviations of the orofacial anthropometric measurements for the control and case groups were calculated and are shown in Table 1.

**Table 1:** Arithmetic mean and standard deviation of orofacial anthropometric measurements.

Orofacial anthropometric measurements	Group		*p*‐Value	Shapiro–Wilk	
	Control	Case		Normality test	
Upper lip	17.91 ± 2.67	21.50 ± 2.77	** ^∗^ *0.002***	0.498	0.882
Lower lip	32.42 ± 7.20	27.97 ± 6.27	0.104	0.298	0.127
Filter	11.47 ± 2.28	9.99 ± 1.30	0.058	0.126	0.872
Upper third of the face	44.73 ± 8.02	43.66 ± 10.03	0.761	0.828	0.888
Middle third of the face	52.26 ± 4.63	48.68 ± 5.22	0.071	0.597	0.552
Lower third of the face	48.59 ± 8.38	50.03 ± 7.44	0.644	0.220	0.352
Distance between ex‐Rch	58.91 ± 3.69	59.09 ± 3.36	0.898	0.815	0.805
Distance between ex‐Lch	58.81 ± 3.29	60.57 ± 3.35	0.185	0.205	0.205
TMF/TIF	1.10 ± 0.17	0.99 ± 0.21	0.153	0.493	0.200
LS/LI	0.57 ± 0.12	0.80 ± 0.20	** ^∗^ *0.001***	0.200	0.724
TSF/TMF	0.86 ± 0.16	0.89 ± 0.19	0.601	0.154	0.428

*Note:* Values in bold indicate statistically significant differences (*p* < 0.05), Student’s *t*‐test. *Source:* Survey data.

^∗^ *p*  < 0.05, Student’s *t*‐test.

Among the anthropometric measurements, a statistically significant difference was observed for upper lip height (sn‐sto), with higher mean values in the CZS group (21.50 ± 2.77) compared to the control group (17.91 ± 2.67) (*p* = 0.002). Similarly, the ratio between the upper lip and lower lip (UL/LL) was significantly greater in the experimental group (0.80 ± 0.20) than in the control group (0.57 ± 0.12) (*p* = 0.001), indicating altered labial proportions in children with CZS. No statistically significant differences were observed for the remaining anthropometric measurements between the groups (*p*  > 0.05).

## 4. Discussion

Recent studies by Carvalho et al. [14] and Freitas et al. [16] emphasize that children with ZIKV congenital syndrome exhibit malformations related to the stomatognathic system. This underscores the importance of evaluating craniofacial growth in children with microcephaly resulting from this syndrome. Due to constraints that make specific radiographic examinations impractical, we opted for an anthropometric approach to measure growth‐related parameters in these children.

Quantitative assessment of craniofacial morphology plays an important role in clinical documentation, research, and the study of craniofacial syndromes and developmental abnormalities [17]. Several methods have been described for this purpose, including anthropometry, cephalometric analysis, and, more recently, three‐dimensional imaging techniques such as facial scanning [13].

Cephalometric analysis has been widely used for the evaluation of craniofacial growth and for orthodontic and orthopedic treatment planning [16]. However, this method requires radiographic imaging, which involves exposure to ionizing radiation, depends on specialized equipment, and requires appropriate patient positioning. In addition, image magnification and distortion may influence measurement accuracy [17].

These aspects may limit its application in pediatric populations and in individuals with craniofacial syndromes, who may have difficulty remaining still during radiographic examinations. In contrast, direct anthropometry represents a practical alternative for craniofacial assessment, particularly in clinical and field settings. This technique does not require ionizing radiation, has relatively low cost, and can be applied to children and individuals with special healthcare needs [16, 17].

When studying orofacial morphology, it is essential to use standard population references corresponding to racial backgrounds as significant morphometric differences have been reported in research involving populations of different races [17]. In this context, Farkas and Deutsch [15] noted that the validity of regular measurements depends on comparison with norms from the appropriate population. The craniofacial differences observed among populations of various races demonstrate that it is inappropriate to apply a standardized norm when establishing an esthetic ideal for different racial and ethnic groups. For this reason, the present study conducted an anthropometric examination of a control group of normosystemic children from the Ceará region, Brazil.

In the present study, the results of anthropometric measurements, which showed a statistically significant difference between the study and control groups, were the mean upper lip height and the upper‐to‐lower lip ratio. Although anthropometry is a viable approach for this research, the literature lacks anthropometric studies involving children with medical conditions, such as CZS.

Considering that most children with CZS are mouth breathers, a study aimed at comparing anthropometric measurements and orofacial proportions between mouth‐breathing children and children without speech complaints by age found differences between the two groups in the measurements of the upper lip, lower lip, lower third of the face, and sides of the face. Additionally, the groups showed differences in the proportions between the upper and lower lips, as well as between the middle and lower thirds of the face [18]. These findings corroborate those of the present study regarding the measurement of the upper lip and the proportion between the upper and lower lips, which also showed statistically significant differences between the two groups.

No statistically significant differences were found between the groups when comparing the means of the face thirds. However, slightly higher values were observed in the experimental group in the lower third of the face, suggesting a tendency toward greater differences. Considering this disproportion and the fact that patients with CZS are mouth breathers, it is believed that the increase in the lower third of the face may be related to their breathing pattern. This is because individuals with a tendency toward vertical growth are predisposed to altered respiratory patterns due to muscle weakness and an increased vertical space in the oral cavity, which may occur in these cases [19].

According to Costa et al. [20], the lack of lip contact during breathing can lead to myofunctional changes. The upper lip may exhibit variations such as shortening, retraction, and alterations in tone, which, in turn, result in motor dysfunction of the lip. The opposite was observed in the children in the experimental group, who showed an increase in upper lip size. No significant difference was found between the groups regarding lower lip size. A correlation was observed between the distances measured from the external corners of the right and left eyes to the upper lip. However, this correlation was not evident in the experimental group as facial growth was compromised by malformations.

Children with microcephaly exhibit altered eating patterns, muscle issues, developmental delays, and abnormal breathing and posture [6]. Recent evidence from studies in typically developing humans demonstrates that facial morphology is strongly influenced by functional factors, particularly the mode of biting and masticatory behavior during postnatal ontogeny. Liang et al. [12] demonstrated that variations in biting mechanics and occlusal function are associated with distinct trajectories of facial growth, highlighting the close relationship between function and craniofacial form in normal development. In this context, altered biting patterns, such as reduced masticatory efficiency, orofacial hypotonia, and atypical oral postures, can lead to disproportionate vertical facial growth and alterations in labial and mandibular morphology. Sequentially present neuromotor impairment, muscle weakness, feeding difficulties, and predominantly oral breathing patterns; it is plausible that these functional disturbances interfere with the normal biomechanical stimuli required for balanced facial growth. Therefore, the altered upper lip height and the increased upper‐to‐lower lip ratio observed in the present study may reflect not only congenital malformations but also the cumulative effects of an atypical biting mode and compromised orofacial function throughout postnatal development. This interpretation situates the findings within the broader framework of functional craniofacial growth and aligns them with current evidence from comparative studies in typically developing populations [12, 14].

According to Farkas and Deutsch [15] and Farkas et al. [17], by the age of one, the lower lip height (sto‐gn) or mandible height reaches 67% of its adult size in both sexes. By approximately 5 years of age, the mandible height (sto‐gn) reaches 87.8% of its adult size. Regarding the growth of the nasolabial region, the height of the philtrum (sn‐ls) reaches 80.2% of its adult size by 1 year of age and ~94.1% by 5 years of age. In the present study, the mean measurements for lower lip height (sto‐gn) and philtrum height (sn‐ls) showed no statistically significant differences between the experimental and control groups. Therefore, it is believed that children with CZS will develop these structures normally in adulthood without significant impairment.

Farkas and Deutsch [15] and Farkas et al. [17] also report that by 1 year of age, the upper lip height (sn‐sto) reaches 81.6% of its adult size, and by 5 years of age, its development increases to 93%. In the present study, the mean upper lip height measurement (sn‐sto) showed a statistically significant difference between the case and control groups, suggesting that children with CZS exhibit an altered growth pattern of this structure in adulthood, which could potentially lead to significant changes in adulthood.

This study has some limitations that should be considered when interpreting the findings. First, the sample size was relatively small, which is largely related to the low prevalence of congenital Zika syndrome and the difficulty in recruiting eligible participants. In addition, the use of direct anthropometry may be subject to measurement variability depending on examiner experience and patient cooperation. Despite these limitations, the use of standardized anthropometric procedures allowed an objective evaluation of facial morphology in this population.

From a clinical perspective, the finding of an increased upper lip height and altered upper‐to‐lower lip ratio in children with CZS may have practical implications. These features can influence lip seal, oral competence, and breathing patterns, which are critical aspects in orofacial myofunctional therapy and speech‐language pathology. Recognizing these morphological tendencies may help guide multidisciplinary care, including early myofunctional interventions and the monitoring of facial growth and esthetics during childhood.

## 5. Conclusion

Within the limitations of this cross‐sectional study, children with CZS showed a statistically significant increase in upper lip height and a higher upper‐to‐lower lip ratio than age‐matched controls. These findings suggest that CZS may influence perioral soft‐tissue development, affecting orofacial function and balance. Further studies with larger samples and longitudinal follow‐up are needed to confirm these preliminary observations and clarify their clinical implications.

## Funding

The authors received no specific funding for this study.

## Conflicts of Interest

The authors declare no conflicts of interest.

## Supporting Information

Additional supporting information can be found online in the Supporting Information section.

## Supporting information


**Supporting Information** The Supporting Information provides individual orofacial anthropometric measurements and calculated facial proportions for the participants included in the study. Files S1 and S2 present the anthropometric measurements and corresponding facial proportions of children with congenital Zika virus syndrome, respectively. Files S3 and S4 present the corresponding anthropometric measurements and facial proportions of normosystemic children, respectively.

## Data Availability

Data sharing is not applicable to this article as no datasets were generated or analyzed during the current study.
