# Supplementary material for: Facial Morphology Assessment in Brazilian Children With Congenital Zika Virus Syndrome: A Cross‐Sectional Anthropometric Study
Source: Int J Dent. 2026 Sep 28;2026:9978691. doi: 10.1155/ijod/9978691 (PMC13620399; doi:10.1155/ijod/9978691)
Supplement: Supplementary file 1 [file IJOD-2026-9978691-s001.docx]

**Supplementary file 1.** Orofacial anthropometric measurements of CZS patients.

| Patients | Upper lip  (mm) | Lower lip  (mm) | Filter (mm) | Upper third of the face  (mm) | Middle third of the face  (mm) | Lower third of the face  (mm) | Distance between ex and Rch  (mm) | Distance between ex and Lch (mm) |  |
| --- | --- | --- | --- | --- | --- | --- | --- | --- | --- |
| Patient 1 | 22.5 | 28.5 | 11.2 | 55 | 47.6 | 51.2 | 62.2 | 62.2 | |
| Patient 2 | 24.7 | 27.4 | 12.1 | 30.3 | 49.3 | 51.8 | 65.4 | 64.5 | |
| Patient 3 | 20 | 24.4 | 9.7 | 36.2 | 40.3 | 45.3 | 54 | 57.3 | |
| Patient 4 | 20 | 24.5 | 8.6 | 50.2 | 48.7 | 42.6 | 60.8 | 62.3 | |
| Patient 5 | 23.7 | 24 | 10.2 | 46.9 | 41.7 | 52.8 | 58 | 62.2 | |
| Patient 6 | 21.8 | 38.5 | 11.7 | 36.1 | 52.7 | 57.2 | 60.1 | 59.5 | |
| Patient 7 | 26.8 | 21.3 | 8.9 | 43.1 | 54 | 39.4 | 58.2 | 59.6 | |
| Patient 8 | 19.9 | 27.5 | 9.9 | 28.9 | 42.2 | 53 | 59.1 | 64.8 | |
| Patient 9 | 17.2 | 21.3 | 8.9 | 43.1 | 54 | 39.4 | 58.2 | 60.8 | |
| Patient 10 | 23.2 | 26.6 | 11 | 63.6 | 56.5 | 51.6 | 62 | 62.2 | |
| Patient 11  Patient 12 | 19.4  18.8 | 41.6  30 | 7.9  9.8 | 42.8  47.7 | 47.2  49.9 | 65.2  50.9 | 57.6  53.5 | 58.8  52.6 | |

Source: Survey data

**Supplementary file 2.** The arithmetic means of the orofacial anthropometric measurements of patients with CZS.

| Patients | TMF/TIF | LS/LI | TSF/TMF |
| --- | --- | --- | --- |
| Patient 1 | 0.92 | 0.78 | 1.15 |
| Patient 2 | 0.95 | 0.9 | 0.61 |
| Patient 3 | 0.88 | 0.81 | 0.89 |
| Patient 4 | 1.14 | 0.81 | 1.03 |
| Patient 5  Patient 6  Patient 7  Patient 8  Patient 9  Patient 10  Patient 11  Patient 12 | 0.78  0.92  1.37  0.79  1.37  1.09  0.72  0.98 | 0.98  0.56  1.25  0.72  0.8  0.87  0.46  0.62 | 1.12  0.68  0.79  0.68  0.79  1.12  0.9  0.95 |

Source: Survey data

**Supplementary file 3.** Orofacial anthropometric measurements of normosystemic patients.

| Patients | Upper lip  (mm) | Lower lip  (mm) | Filter (mm) | Upper third of the face  (mm) | Middle third of the face  (mm) | Lower third of the face  (mm) | Distance between ex and Rch  (mm) | Distance between ex and Lch  (mm) |
| --- | --- | --- | --- | --- | --- | --- | --- | --- |
| Patient 1 | 21.8 | 26.5 | 14 | 46.7 | 51.3 | 47.7 | 58.2 | 58.7 |
| Patient 2 | 17.4 | 26 | 9.7 | 44.5 | 47.8 | 41.4 | 58 | 58 |
| Patient 3 | 16.7 | 35.2 | 9.5 | 41.1 | 56.2 | 53.7 | 51.3 | 55.5 |
| Patient 4 | 20.4 | 26.08 | 11.1 | 57 | 53.5 | 46.2 | 63.5 | 63.7 |
| Patient 5 | 15.2 | 24.4 | 11 | 26.7 | 41.6 | 27.3 | 54.2 | 53.2 |
| Patient 6 | 17.9 | 28.7 | 10.8 | 42.6 | 58.9 | 48.3 | 61.4 | 62 |
| Patient 7 | 17.6 | 35.1 | 9.2 | 40.5 | 58.4 | 51.2 | 57.8 | 58.4 |
| Patient 8 | 15.1 | 30.1 | 11.3 | 53.3 | 48.7 | 48.2 | 57 | 57 |
| Patient 9 | 16.9 | 33.4 | 12.1 | 37 | 55.5 | 52.7 | 61.2 | 62.7 |
| Patient 10 | 16.2 | 29.7 | 12.5 | 46 | 55 | 46.1 | 55.2 | 55.4 |
| Patient 11 | 16.8 | 38.6 | 10.8 | 52.3 | 49.6 | 58.5 | 58.1 | 56.2 |
| Patient 12  Patient 13  Patient 14  Patient 15 | 18.9  19.7  24  14 | 44.6  41.6  43.9  22.4 | 12.5  18.1  10.1  9.3 | 44.9  53.3  49.9  35.1 | 52.6  50.3  55.8  48.7 | 54.9  54.7  59.9  38 | 62.6  64.8  61.9  58.5 | 62.7  57.8  63.3  57.6 |

**Supplementary file 4.** The arithmetic means of the orofacial anthropometric measurements of patients with normosystemic

| Patients | TMF/TIF | LS/LI | TSF/TMF |
| --- | --- | --- | --- |
| Patient 1 | 1.07 | 0.82 | 0.91 |
| Patient 2 | 1.15 | 0.66 | 0.93 |
| Patient 3 | 1.04 | 0.47 | 0.73 |
| Patient 4 | 1.15 | 0.78 | 1.06 |
| Patient 5  Patient 6  Patient 7  Patient 8  Patient 9  Patient 10  Patient 11  Patient 12  Patient 13  Patient 14  Patient 15 | 1.52  1.21  1.14  1.01  1.05  1.19  0.84  0.95  0.91  0.93  1.28 | 0.62  0.62  0.50  0.50  0.50  0.54  0.43  0.42  0.47  0.54  0.62 | 0.64  0.72  0.69  1.09  0.66  0.83  1.05  0.85  1.05  0.89  0.72 |

Source: Survey data
